# Trichostatin A-modified vaccine provides superior protection against ovarian cancer formation and development

**DOI:** 10.1590/1414-431X2024e12874

**Published:** 2024-05-17

**Authors:** Yingwei Liu, Tao Yi, Shenglan Meng, Xia Zhao, Xiancheng Chen, Yanna Zhang

**Affiliations:** 1 Department of Gynecology, First Affiliated Hospital of Chongqing Medical University, Chongqing, China; 2 National Key Laboratory of Biotherapy and Cancer Center, West China Hospital, Sichuan University, Chengdu, Sichuan, China; 3 Department of Gynecology & Obstetrics, West China Second Hospital, Sichuan University, Chengdu, Sichuan, China; 4 Department of Blood Transfusion, Sichuan Provincial People’s Hospital, University of Electronic Science and Technology of China, Chengdu, Sichuan, China

**Keywords:** Epigenetic vaccine, Bioprevention, Tumorigenesis, Ovarian cancer, Trichostatin A

## Abstract

More attention has been paid to immunotherapy for ovarian cancer and the development of tumor vaccines. We developed a trichostatin A (TSA)-modified tumor vaccine with potent immunomodulating activities that can inhibit the growth of ovarian cancer in rats and stimulate immune cell response *in vivo*. TSA-treated Nutu-19 cells inactivated by X-ray radiation were used as a tumor vaccine in rat ovarian cancer models. Prophylactic and therapeutic experiments were performed with TSA-modified tumor vaccine in rats. Flow cytometry and ELISpot assays were conducted to assess immune response. Immune cell expression in the spleen and thymus were detected by immunohistochemical staining. GM-CSF, IL-7, IL-17, LIF, LIX, KC, MCP-1, MIP-2, M-CSF, IP-10/CXCL10, MIG/CXCL9, RANTES, IL-4, IFN-γ, and VEGF expressions were detected with Milliplex Map Magnetic Bead Panel immunoassay. TSA vaccination in therapeutic and prophylactic models could effectively stimulate innate immunity and boost the adaptive humoral and cell-mediated immune responses to inhibit the growth and tumorigenesis of ovarian cancer. This vaccine stimulated the thymus into reactivating status and enhanced infiltrating lymphocytes in tumor-bearing rats. The expression of key immunoregulatory factors were upregulated in the vaccine group. The intensities of infiltrating CD4+ and CD8+ T cells and NK cells were significantly increased in the vaccine group compared to the control group (P<0.05). This protection was mainly dependent on the IFN-γ pathway and, to a much lesser extent, by the IL-4 pathway. The tumor cells only irradiated by X-ray as the control group still showed a slight immune effect, indicating that irradiated cells may also cause certain immune antigen exposure, but the efficacy was not as significant as that of the TSA-modified tumor vaccine. Our study revealed the potential application of the TSA-modified tumor vaccine as a novel tumor vaccine against tumor refractoriness and growth. These findings offer a better understanding of the immunomodulatory effects of the vaccine against latent tumorigenesis and progression. This tumor vaccine therapy may increase antigen exposure, synergistically activate the immune system, and ultimately improve remission rates. A vaccine strategy designed to induce effective tumor immune response is being considered for cancer immunotherapy.

## Introduction

Ovarian cancer (OC) is fatal due to the absence of specific symptoms and timely diagnosis. More than 70% of patients are firstly diagnosed with stage III and IV tumors, which generally have a poor prognosis and few effective treatment options. The five-year survival rates of stage III and IV OC are 35 and 20%, respectively, and cancer recurrence occurs in 60-70% of patients with optimal debulking operation (1). Platinum-taxane maintenance is still the first therapeutic option for OC. Approved maintenance therapy of bevacizumab or PARP inhibitors has shown some efficacy to prolong progression-free survival (PFS) but not overall survival (OS); most of the patients also die from their disease despite response to first-line therapy (2). Thus, it is necessary to discover more therapeutic approaches for treating OC patients.

Increasing evidence suggests that OC is considered an immunogenic tumor and supports the efficacy of immune therapy (3,4). Different treatment strategies have been proposed, some of which are being clinically used. The dramatic advances in cellular immunotherapy have created new opportunities in the treatment of OC. Infiltrating CD8+ T cells have been reported to affect the prognosis of OC patients (5).

Previous research has confirmed that OC is indeed an immunogenic tumor (6,7). Some OC antigens have been identified and analyzed, including HER2/neu, p53, IGF-binding protein 2, folate receptor α, mucins, NY-ESO-1, and epithelial cell adhesion molecule. These antigens can provoke a response in OC patients (6). Cancer vaccines have emerged as a promising immunotherapeutic approach for the treatment of OC (8,9). The main purpose of cancer vaccines is to initiate tumor suppression by activating tumor-specific T lymphocytes *in vivo* (7). Cell-mediated cancer vaccines include allogeneic or autologous tumor cells that can be altered by chemical agents *in vitro* (10). However, the practical application of an unmodified tumor cell-based vaccine is limited due to its immunogenicity, and deficiency of OC antigens with favorable expression patterns is also observed for tumor vaccines (11).

The production of personalized cancer vaccines made from autologous tumor cells might benefit from mechanisms that enhance immunogenicity (12). Evidence that cancer can induce tumor-specific immune responses has driven the development of therapeutic vaccines (13). Our previous study indicated that histone deacetylase inhibitor (HDACi) trichostatin A (TSA) could mediate apoptosis of ovarian tumor cells in a concentration-dependent manner (14).

Acetylation of histones and histone deacetylases (HDACs) has been extensively studied, which can regulate gene repression globally or specifically (11). The acetylation status in cells is controlled by the balance between histone deacetylases and histone acetyltransferases. The HDACi TSA has been shown to regulate the expression of ∼5% of the genome (15). HDACi can induce the expression of immune-related molecules in cancer cells, and HDACi-treated cancer cells can trigger immune responses both *in vivo* and *in vitro* (16,17). The HDACi agents have been shown to provoke antitumor immunity in melanoma mouse model. MHC class II and costimulatory molecules B7-1/2 and CD40 are activated by TSA on the cell tumor plasma in a dose-dependent manner. Upon epigenetic activation, MHC class II is transported from the plasma to the cell surface and converts the cell to an antigen presenting cell (APC) for class II-peptide and protein presentation. Tumor cells treated with chromatin modification agents may be potential epigenetic vaccines (16). However, the mechanism underlying the immune responses of these vaccines remains poorly understood. The conversion of tumor cells to APCs by HDACi treatment can provide additional pathways to overcome OC. The effectiveness of epigenetic vaccines may be derived from cross-presentation, which is regulated by HDACi-induced apoptosis. Epigenetic activation of immune genes also contributes to direct antigen presentation by cancer cells (18). In addition, the effects of these vaccines are varied across different types of cancers. We speculated that the immune response triggered by TSA could be used as a modification of cellular vaccines for the treatment of tumors.

In this study, the therapeutic effects of tumor cell-based vaccines in combination with TSA were determined using a rat model. We hypothesized that the irradiated cell-based tumor vaccine treated with TSA can exhibit potential antitumor effects on OC in vaccinated rats. The present study aimed to determine whether the epigenetic modifications of chromatin to reverse gene silencing can facilitate the development of an effective cancer vaccine.

## Material and Methods

### Materials

The rat ovarian tumor cell line NuTu-19 was supplied by ATCC (USA) and kept at the State Key Laboratory of Biotherapy of Human Diseases (West China Hospital of Sichuan University, China). NuTu-19 was initiated from a poorly differentiated adenocarcinoma that arose in a female athymic mouse injected with Fischer 344 ovarian surface epithelial cells (15). Cells were cultured in RPMI 1640 (Gibco Life Technologies, USA) with 10% FBS, 2 mM L-glutamine, 100 g/mL streptomycin, 100 units/mL penicillin, and maintained at 37°C and 5% CO_2_. Fischer 344 rats (female, pathogen-free, 100-120 g) were obtained from the West China Experimental Animal Center (China) and housed in a pathogen-free animal facility at the University of Sichuan University. Food and water were provided *ad libitum*. Trichostatin A (TSA) and DMSO were purchased from Sigma (USA). TSA was dissolved in DMSO (up to 0.1%). Cells were treated with 200, 500, or 800 nM of TSA for 48 h before analysis. Cells were X-ray irradiated with 120 Gy to remain metabolically active without proliferation, and then frozen for further use.

### Immunotherapy and tumor models

**Figure 1:**
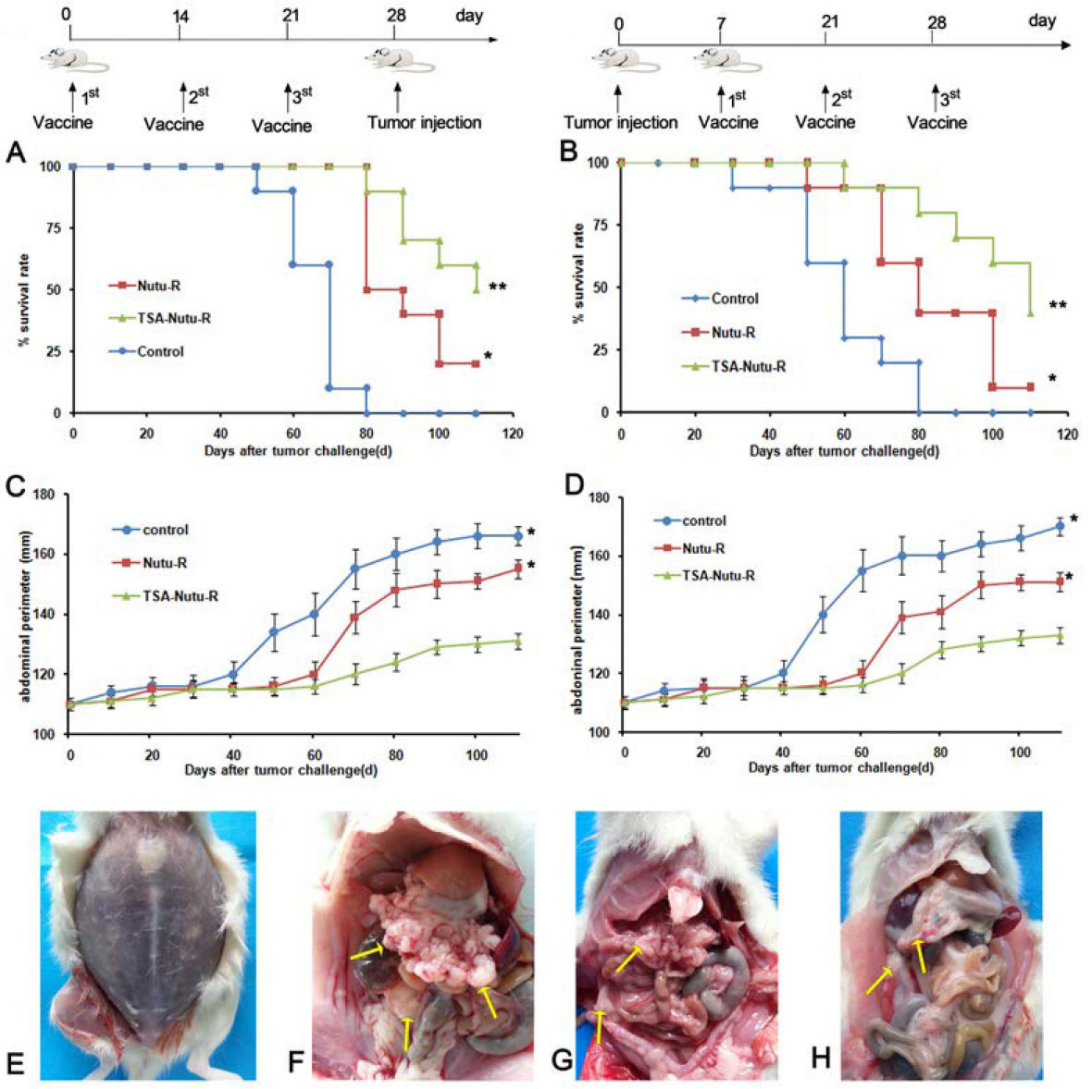
Trichostatin A (TSA)-Nutu-R vaccine inhibits the ovarian cancer tumorigenesis and growth. **A** and **C**, rats (n=10) were treated with 500 nM TSA-Nutu-R vaccine, Nutu-R vaccine (on days 0, 14, and 21), or left untreated (control group) and challenged with 1×10^6^ Nutu-19 cells (on day 28) in the prophylactic model. **B** and **D**, Nutu-19 cell-bearing rats (n=10) were treated with 500 nM TSA-Nutu-R vaccine, Nutu-R vaccine, or Control (normal saline) (on days 7, 21, and 28) in the therapeutic model. **E**, After tumor cell inoculation, rats in the control group had abdominal distension and more bloody ascites in the abdominal cavity. **F**, In the control group, there were many tumor nodules in the abdominal cavity of rats, which were distributed in uterus, double adnexa, omentum, abdominal wall, liver, and spleen (arrows). **G**, Full peritoneal cavity of a rat from the therapeutic model treated with Nutu-R vaccine and an open view of the cavity with some metastatic nodes and tumor masses (arrows) (after aspirating 55 mL of ascites). **H**, Full peritoneal cavity of a rat from the therapeutic model treated with TSA-Nutu-R vaccine and an open view of the cavity with fewer metastatic nodes and tumor masses. Ten rats were included in each group. The animals were euthanized on day 110.

Rats were fed with a rodent diet and autoclaved reverse osmosis water for 2 to 3 weeks and acclimated to their living environments prior to study initiation. For prophylactic experiments (Figure 1A), NuTu-19 cells were collected with 0.25% trypsin-EDTA (Gibco), and TSA-treated and irradiated cells were subcutaneously injected (left flank) into Fischer 344 rats at days 0, 14, and 21, and then intraperitoneally challenged with viable NuTu-19 cells (10^6^ cells/mL in PBA) at day 28. All rats were monitored for abdominal perimeter and survival. For the therapeutic model (Figure 1B), 7 days after peritoneal cavity tumor implantation, rats were vaccinated with TSA-treated and irradiated (120 Gy) NuTu-19 cells in the left flank. Immunotherapy was performed at days 7, 21, and 28 after tumor vaccination. Tumor-bearing control rats were either treated with irradiated NuTu-19 cells or left untreated and received normal saline (NS). Tumor-free rats were observed for another 110 days. All rats were under careful observation to examine tumor formation and metastasis, as well as modulation in physical appearance or behavior. Ethical approval was obtained from the Ethics Review Committee for Animal Experimentation of Sichuan University.

Humane endpoints take the following into consideration: abdominal girth, animal weight, body condition score, animal mobility, and activity. We checked the animals twice a day. If the above conditions were found to be deteriorated or an animal was dying, the rat was euthanized at the end of its life and relevant experiments were conducted. If no serious complications occurred after 110 days, the animals were euthanized on day 110.

### Flow cytometry

To determine whether the TSA-treated and irradiated (120 Gy) NuTu-19 cells vaccine (TSA-NuTu-R vaccine) can induce serum variation after immunization, circulating peripheral blood serum from both experimental and control groups were harvested at day 7 after the third immunization. All specimens were examined by flow cytometry for the potential of binding to NuTu-19 OC cells. Peripheral blood (PB: 1 mL/each rat) was collected from different groups (control, NuTu-R vaccine, TSA-NuTu-R vaccine) at day 7 after the last immunization. Then, 10 μL peripheral blood serum and 1×10^6^ NuTu-19 OC cells were added into the tubes, fully blended, and then placed in the dark for 60 min. Afterwards, the supernatant was removed and mixed with 2 mL PBS (1 g/L sodium azide), shaken, fully mixed, and centrifuged (200 *g*, 5 min, 20°C). Finally, FITC-secondary antibody was blended into the tubes for flow cytometry assay (Becton Dickinson, USA).

### *In vitro* cytotoxicity assay

To determine the potential cytotoxicity of cytotoxic T lymphocyte (CTL), the 4-h Chromium-51 release assay was conducted according to a previous report (16). Splenocytes obtained from the TSA-NuTu-R vaccine immunized rats or control rats were exposed to ammonium chloride potassium lysing buffer for erythrocyte depletion. For the preparation of the T cell-enriched fraction, these splenocytes were incubated for 90 min in complete medium. After gentle shaking, the non-adherent cells were incubated on a nylon wool column. Then, 100 µL of effector cells and 51Cr-labeled target cells at different ratios (1:10, 1:20, and 1:40) were added into a microtiter plate and then incubated at 37°C for 4 h. The supernatants were collected, and the released radioactivity was detected using a gamma counter (LKB Wallac, Finland). The following equation was employed: cytotoxicity (%) = [(experimental release - spontaneous release) / (maximum release - spontaneous release)] × 100.

### *In vitro* analyses of systemic immune response

The Dual-Color IFN-γ/IL-4 ELISpot Kit (R&D, USA ) was used to monitor the primary and cross responses of immunized rats to TSA-treated and irradiated NuTu-19 cells and tumor cells, respectively. Briefly, the rats were immunized with TSA-treated and irradiated NuTu-19 cells as described above and sacrificed 2 weeks after the last boost vaccination. The spleen was collected before splenocyte isolation. Assays using 3×10^3^ X-ray-irradiated tumor cells as stimulator cells and 10^5^ recipient splenocytes as responder cells were carried out. An ELISpot plate reader (CTL Analyzers; Cellular Technology, USA) was used to automatically enumerate the spots for further analysis.

### Adoptive cell transfer

On day 7 following the third immunization, serum and splenocytes were isolated aseptically from the treated or naive Fischer 344 rats for passive serum/cellular therapy. The donor splenocytes (2×10^7^) were intravenously injected into the recipient rats for two consecutive days from the second day after OC inoculation (17). Tumor-bearing rats were intravenously injected with 0.1 mL serum on days 1-10 after immunization with TSA-treated and irradiated NuTu-19 cells. To assess the effects of passive serum therapy on OC, 5-7-week-old female rats received subcutaneous inoculation of 1×10^6^ NuTu-19 cells at day 0. Tumor-bearing rats were treated with passive serum therapy as cited above.

### Immunohistochemical staining

Tumor samples, heart, lung, spleen, liver, kidney, and thymus were fixed with formalin, followed by dehydration with a graded series of alcohols. After immersing in paraffin wax, the specimens were sliced and stained with hematoxylin and eosin (H&E). Cancer tissues for immunohistochemistry were cut (5 μm), mounted on slides, deparaffinized in xylene, and rehydrated through graded ethanol. Antigen retrieval was accomplished by steam heating. Endogenous peroxidase was blocked by 3% H_2_O_2_ for 30 min. The sections were individually exposed to anti-CD49b/NK1.1 (BioLegend, USA), anti-CD4, anti-CD8, anti-CD3, anti-CD24, antiFOXP3, anti-PAKT, anti-AKT-2, and anti-RhoB (1:200 dilution; Abcam, USA) overnight at 4°C. After incubation with biotinylated anti-mouse antibodies, the sections were incubated again with streptavidin-biotinylated peroxidase complex. DAB chromogen was used to develop the peroxidase color reaction. Lastly, Meyer's hematoxylin was used to counterstain all sections. The infiltrated lymphocytes were examined and counted using a microscope (400× magnification, Nikon, Japan). ImageJ (NIH, USA) was used for immunohistochemical analysis, and IHC Profiler was used to automatically score the staining of samples. The different intensity percentages of positive cells were estimated at ×400 magnification. The intensity of cell stain was given a score on a scale of 0-3, with 0=negative, 1=light, 2=moderate, and 3=intense. The intensity percentage of positive cells is reported as the ratio of differently intense positive cells.

### Magnetic bead microarray

Non-necrotic fresh tumors were cut into small parts, homogenized in liquid nitrogen, emulsified by ultrasonication, isolated with ice-cold RIPA buffer at a ratio of 100 mg:1 mL, and then passed through a fine mesh sieve (Bellco Glass, USA). Total protein or blood sera of vaccinated rats was concentrated to 12 mg/mL, followed by detection for GM-CSF, IL-7, IL-17, LIF, LIX, KC, MCP-1, MIP-2, M-CSF, IP-10/CXCL10, MIG/CXCL9, RANTES, IL-4, IFN-γ, and VEGF expression with Milliplex Map Magnetic Bead Panel Immunoassay (Millipore, USA) using Luminex 200 magnetic bead microarray analytical system (Luminex Corp., USA). Each specimen was run in duplicate.

### Determination of adverse effects

Rats immunized with these vaccines were evaluated, especially for cytotoxicity, for at least 6 months. Gross measures, including behavior, ruffling of fur, weight loss, and life span, were determined. The heart, lung, liver, kidney, spleen, and other tissues were fixed in neutral buffered formalin (10%), embedded in paraffin, sectioned (4-5 mm), and stained with H&E. The stained sections were examined by two pathologists blinded to the experimental arms. Fertility was also evaluated. Briefly, 4 months after the fourth immunization, female rats were allowed to cohabitate with males. The number of pups and days until parturition were recorded. All rats (n=16) were subjected to complete blood count and differential cell count. The neuromuscular performances of control and vaccinated rats were assessed using the wire hang and footprint tests (16).

### Statistical analysis

SPSS software package system was used for all statistical analyses. Numerical data were analyzed by one-way ANOVA plus Tukey *post hoc* test or two-way ANOVA and repeated measures when comparing more than two groups. Ranked data were analyzed by Kruskal-Wallis test plus Bonferroni when comparing more than two groups. Tumor-free survival times among groups were compared via logrank test and Kaplan-Meier method. Numerical values are reported as means±SD. Statistical significance was assumed for P<0.05.

## Results

### TSA-NuTu-R vaccine induced therapeutic and protective antitumor immunity

In the prophylactic model (Figure 1A and C), the rats were immunized with TSA-NuTu-R vaccines, NuTu-R vaccines, or NS on days 0, 14, and 21, and then challenged with tumor cells. Tumors grew progressively in both non-immunized and NuTu-R vaccines-immunized rats but were significantly suppressed in rats immunized with TSA-NuTu-R vaccine (P<0.05). These findings demonstrated that TSA-NuTu-R vaccination can induce antitumor immune responses to attenuate the growth and tumorigenesis of OC in rats.

In the therapeutic model, the rats were treated at day 7 following tumor cell implantation (Figure 1B and D). Treatment with TSA-NuTu-R vaccine on days 7, 21, and 28 significantly increased antitumor activity in OC models (Figure 1B). The survival rate of TSA-NuTu-R vaccine-treated tumor-bearing rats was significantly greater than that of control rats. The growth of OC in both TSA-NuTu-R and NuTu-R groups was significantly retarded compared to the control group (P<0.05).

**Figure 2:**
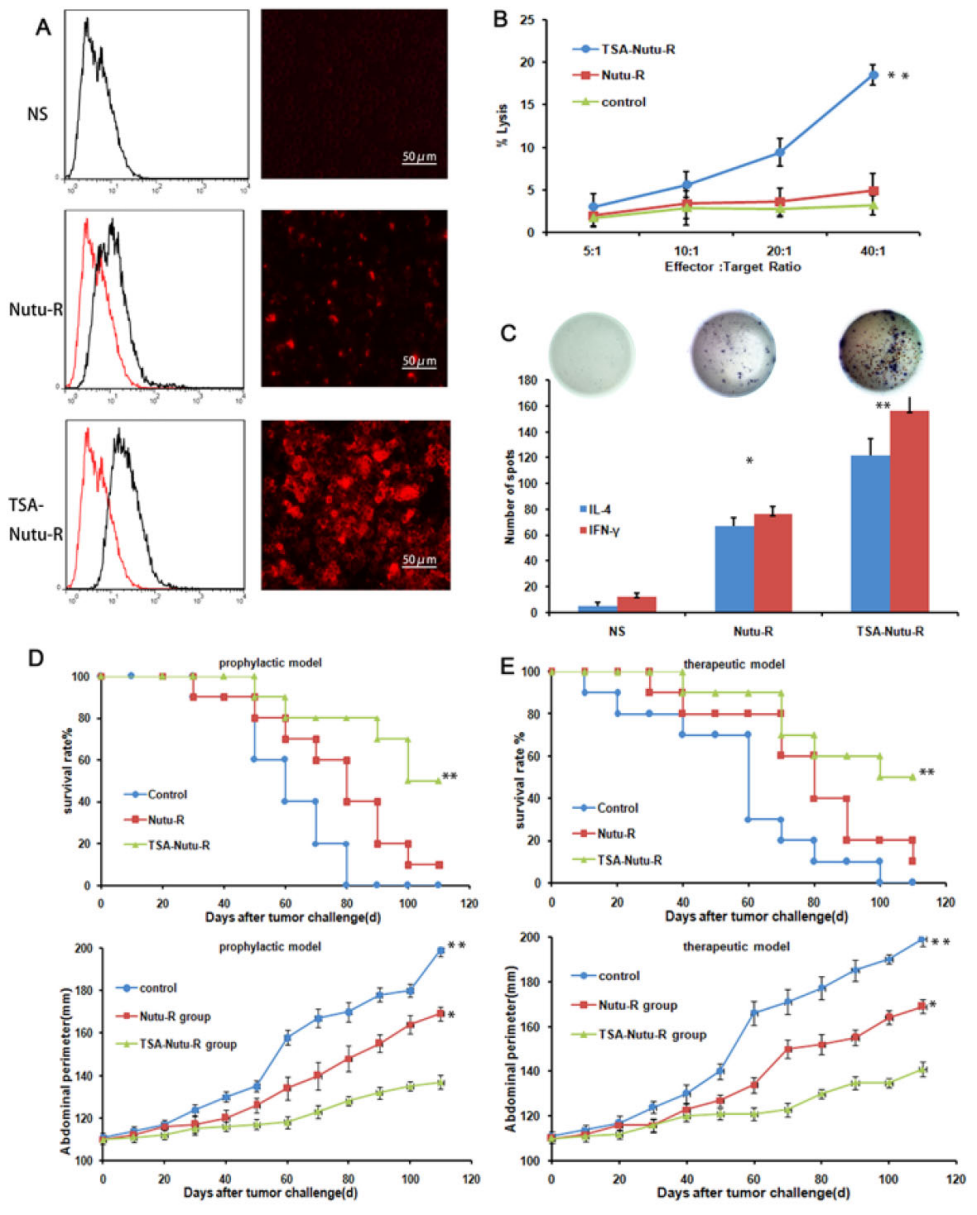
Trichostatin A (TSA-Nutu-R) vaccine inoculation triggers protective responses. **A**, The immunofluorescence assay showed that the sera from the vaccinated rats exhibited more than 70% of luminescence rates for Nutu-19 surface antibody (images, scale bar, 50 μm). The potential of peripheral serum binding to Nutu-19 cells was detected by flow cytometry (graphs). **B**, Antibody-dependent cellular cytotoxicity was determined using the Chromium-51 release assay, which indicates cell lysis. **C**, Dual-color ELISpot assay reveals enhanced releases of interferon (IFN)-γ and interleukin (IL)-4 by recipient hosts splenocytes from TSA-Nutu-R vaccine-inoculated groups but not from other groups (**P<0.01 *vs* controls, ANOVA). The assay was run in three replicates. **D**, In the prophylactic model, TSA-Nutu-R vaccine inhibited tumor formation (measured by abdominal circumference) and increased survival measured every 10 days and monitored for 100 days. **E**, Similar findings were observed in the therapeutic model. Ten rats were included in each group. The animals were euthanized at day 110. Data are reported as means and SD. *P<0.05, **P<0.01 (Kaplan-Meier analysis). NS: normal saline control.

The antitumor effects of the epigenetic vaccine were further assessed in an adoptive cellular/serum therapy model (Figure 2D and E). The results indicated that the growth of OC in the TSA-NuTu-R vaccine group was significantly suppressed after passive serum therapy for 10 consecutive days (P<0.05; Figure 2D). Next, the cells from the immunized rats were passively transferred to the recipient Fischer 334 rats twice a day after OC challenge (Figure 2E). Fischer 334 rats receiving serum from naive rats all succumbed to tumors. On the contrary, serial passive transfer of serum or cells from the TSA-NuTu-R vaccine group markedly suppressed the development of NuTu-19 OC (P<0.05 *vs* control).

No significant toxic effects, such as ruffed fur, weight loss, anorexia, and diarrhea, were observed throughout the entire vaccination process in both therapeutic and preventive models. To assess the potential toxicity of the epigenetic vaccine on vital organs in rats, immunohistochemical staining was carried out. No significant pathological changes were observed in the liver, heart, kidney, and lung of rats after treatment (Supplementary Figure S1).

### TSA-NuTu-R vaccine improved antigen-specific antibody immune responses via induction of potent cytotoxic T lymphocytes (CTL) responses

Flow cytometry analysis revealed that the binding to TSA-NuTu-19 cells was significantly increased in the experimental group compared to the control group (P<0.05; Figure 2A). Consistent with this, immunofluorescence also showed that NuTu-19 cells could bind to the serum isolated from rats immunized with TSA-NuTu-R vaccine, but negative staining was observed in rats immunized with NuTu-R vaccine and the control NS group (Figure 2A). Therefore, it can be inferred that special immune molecules in the immunized-serum are not only generated during the immune response process, but also mobilized to exert a certain role in this response process through which the effect may be perfectly understood.

To evaluate the cellular immune response induced by the TSA-NuTu-R vaccine, CTL activities were evaluated using the Chromium-51 release assay. Splenocytes were isolated and cultured with NuTu-19 cells for 16 h, and subsequently applied as an effector. The results of CTL assay (Figure 2B) were verified according to the ratio of spontaneous release-to-maximum release. The effector in TSA-NuTu-R vaccine rats showed 3.9- and 7.1-fold higher tumor-killing activities (lysis) compared with NuTu-R vaccine and control groups, respectively (P<0.05).

Next, the number of spot-forming cells (SFCs) after *in vitro* stimulation with TSA-NuTu-R vaccine was detected using the ELISPOT assay. In the SFCs, the levels of IFN-γ/IL-4 were significantly increased in the TSA immunization group compared with NuTu-R vaccines or control group (P<0.05). The number of IFN-γ-secreting SFCs in the TSA-NuTu-R vaccine group was 19.8-fold over that of the control group (P<0.05). The number of IL-4-secreting SFCs in TSA-NuTu-R vaccines was 11.4-fold over that of control group (P<0.05; Figure 2C). In the prophylactic model, TSA-Nutu-R vaccine inhibited tumor formation (measured by abdominal circumference) and increased survival measured every 10 days. There were statistical differences in abdominal circumference and survival rate between the experimental group and the control group (P<0.05) (Figure 2D). Similar findings were observed in the therapeutic model (P<0.05) (Figure 2E).

### TSA-NuTu-R vaccine enhanced infiltrating lymphocytes in tumor-bearing rats

**Figure 3:**
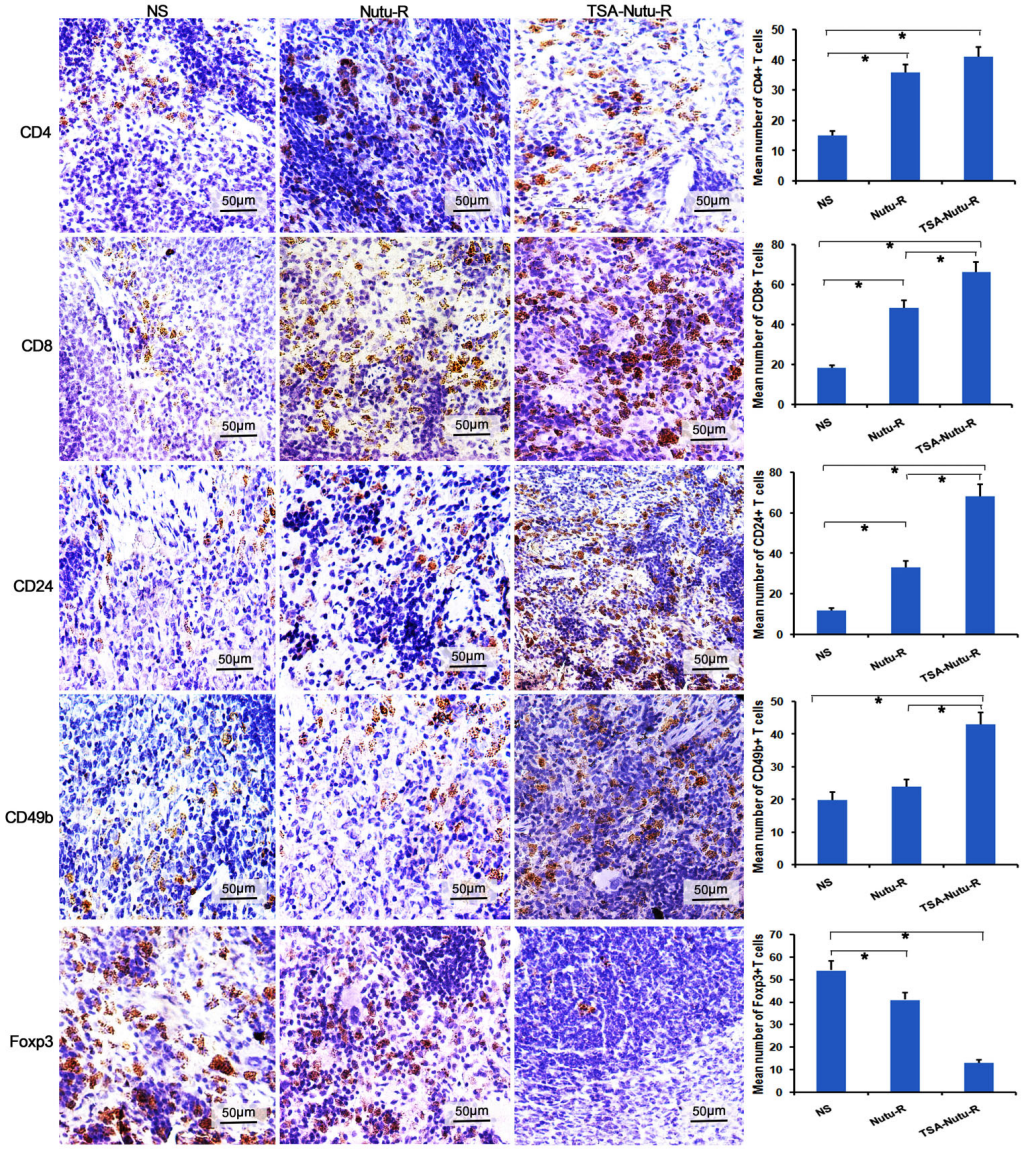
Immunohistochemistry assays show increased CD4+, CD8+, CD24, CD49b, and Foxp3 T cells scattered in spleens of trichostatin A (TSA)-Nutu-R vaccine groups (micrographs at 400× magnification, scale bar 50 μm). Numerous CD25+Foxp3+ T suppressor cells were congregated in spleens, but fewer T suppressor cells were found in the Nutu-R vaccine group and TSA-Nutu-R vaccine groups. Data are reported as means and SD. *P<0.05 (ANOVA).

Tumor-infiltrating lymphocyte (TIL), an important component of solid tumor infiltrate, is believed to regulate host antitumor immunity. Immunofluorescence analysis was conducted to assess TIL in tumor-bearing rats. The intensities of CD4, CD8, Foxp3, CD24, and CD49b infiltrating lymphocytes were analyzed (Figure 3). Notably, the intensities of infiltrating CD4+ and CD8+ T cells were significantly increased in TSA-NuTu-R and NuTu-R vaccine groups compared to the control group (P<0.05). The infiltrating NK cells were significantly higher in the TSA-NuTu-R vaccine group than in the NS group (P<0.05). Moreover, the infiltrating CD4+ and CD8+ T cells and NK cells were abundantly found in the TSA-NuTu-R vaccine group compared with the NuTu-R vaccine and NS groups (P<0.01; Figure 3).

### TSA-NuTu-R vaccine stimulated the thymus into reactivating status

**Figure 4:**
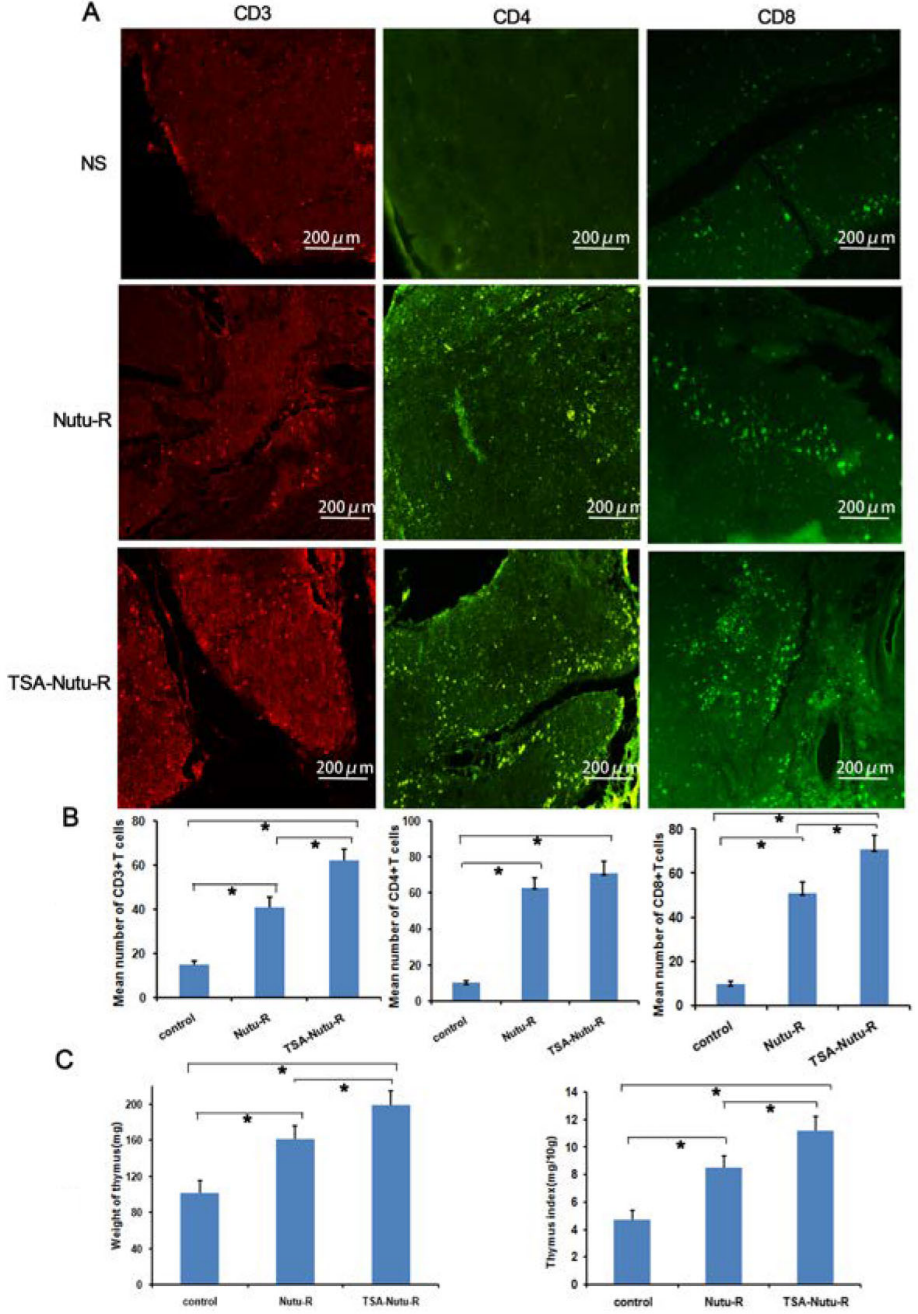
Trichostatin A (TSA)-Nutu-R vaccine stimulated the thymus into a reactivating status. **A** and **B**, A higher number of CD3+, CD4+, and CD8+ T cells were found in the thymus of TSA-Nutu-R vaccine-inoculated groups (scale bar 200 μm). **C**, The thymuses were carefully collected and then weighed immediately to assess the antitumoral efficacy. The average weight of the thymus in the TSA-Nutu-R vaccine group was 186.3±12.8 mg, compared with the Nutu-R vaccine group (164.6±12.1 mg) and control group (91.1±8.7 mg). Data are reported as means and SD. *P<0.05 (ANOVA).

Immunohistochemical and immunofluorescence analyses were conducted to assess the lymphocytes in both spleen and thymus. Figure 3 indicates the types of infiltrating lymphocytes and the data of CD4, CD8, CD24, and CD49b staining. It was found that the densities of CD4^+^, CD8^+^, and CD49b^+^ T cells were all elevated in the spleens of TSA-NuTu-R vaccine group (P<0.05 *vs* other groups). Similarly, the densities of CD3^+^, CD4^+^, and CD8^+^ T cells were also increased in the thymuses of TSA-NuTu-R vaccine group (P<0.05 *vs* other groups) (Figure 4).

### Expression of key immunoregulatory factors was upregulated in TSA-NuTu-R vaccine group

**Figure 5:**
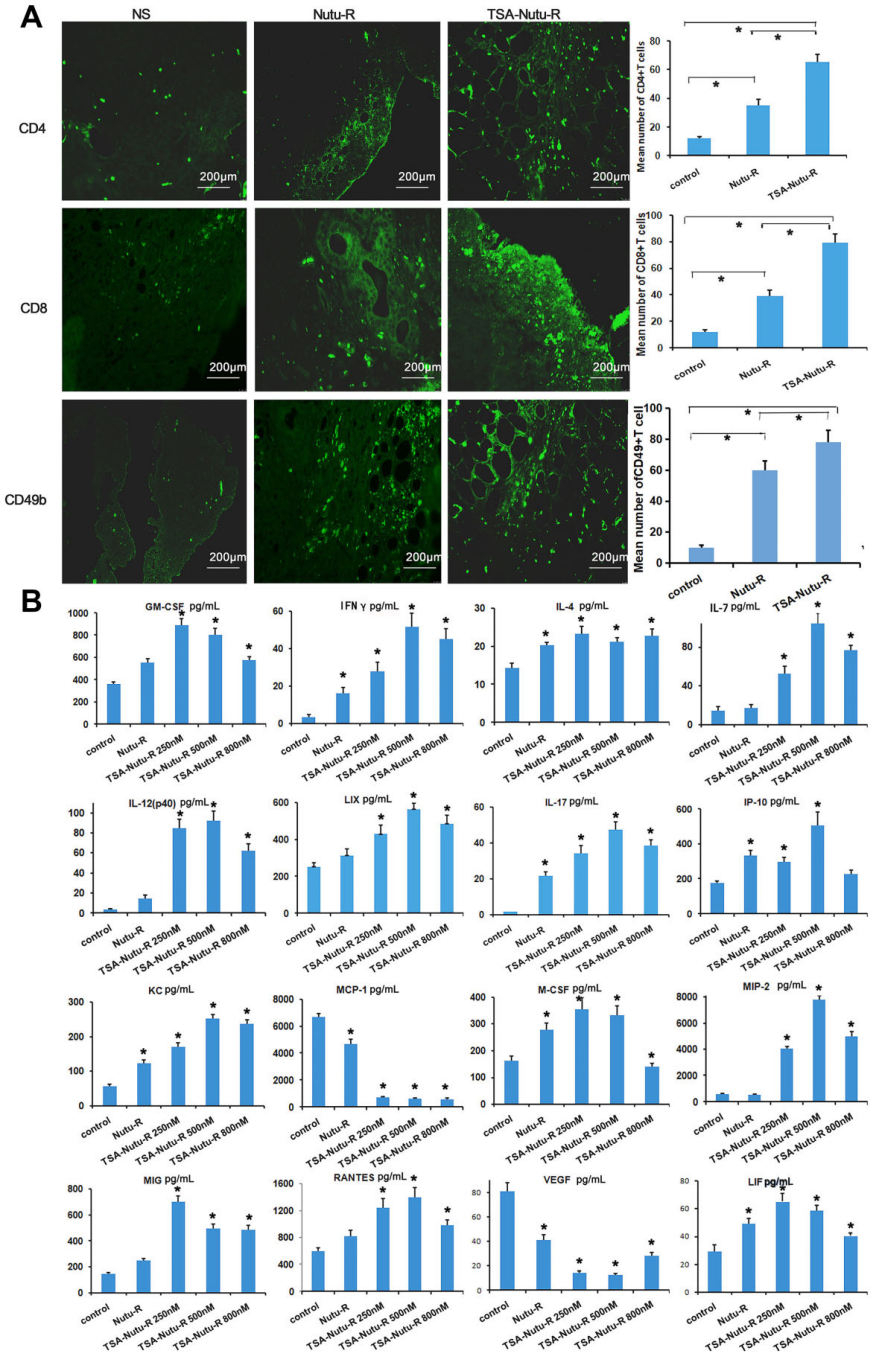
Expression levels of multiple key factors of the immunoregulatory network including cytokines and chemokines in trichostatin A (TSA)-Nutu-R vaccine group were investigated by magnetic bead microarray (scale bar 200 μm). **A**, The expression of invasive CD4+, CD8+, and CD49b+ T lymphocytes in ovarian cancer tissues of rats immunized with TSA-Nutu-R vaccine increased (P<0.05). **B,** The TSA-NuTu-R vaccine group showed an upregulated expression of GM-CSF, IFN-γ, IL-4, IL-7, IL-12, LIX, IL-17, IP-10, KC, M-CSF, MIP-2, MIG, RANTES, and LIF (P<0.05). Concomitant downregulation of MCP-1 and VEGF was also observed (P<0.05). Data are reported as means and SD. *P<0.05 *vs* control (ANOVA).

Expression levels of multiple key factors of the immunoregulatory network including cytokines and chemokines are shown in the TSA-Nutu-R vaccine group (P<0.05, Figure 5A). TSA-NuTu-R vaccine group showed an upregulated expression of GM-CSF, IFN-γ, IL-4, IL-7, IL-12, LIX, IL-17, IP-10, KC, M-CSF, MIP-2, MIG, RANTES, and LIF (P<0.05). Concomitant downregulation of MCP-1 and VEGF was also observed (P<0.05, Figure 5B). These results indicated that TSA-NuTu-R vaccine triggered the immune system and the integration of more than one cytokine and chemokine involved in antitumor immunoregulation, thereby prolonging the survival rates of tumor-bearing rats.

### Activation of PI3/Akt signaling pathway by TSA-NuTu-R vaccine

**Figure 6:**
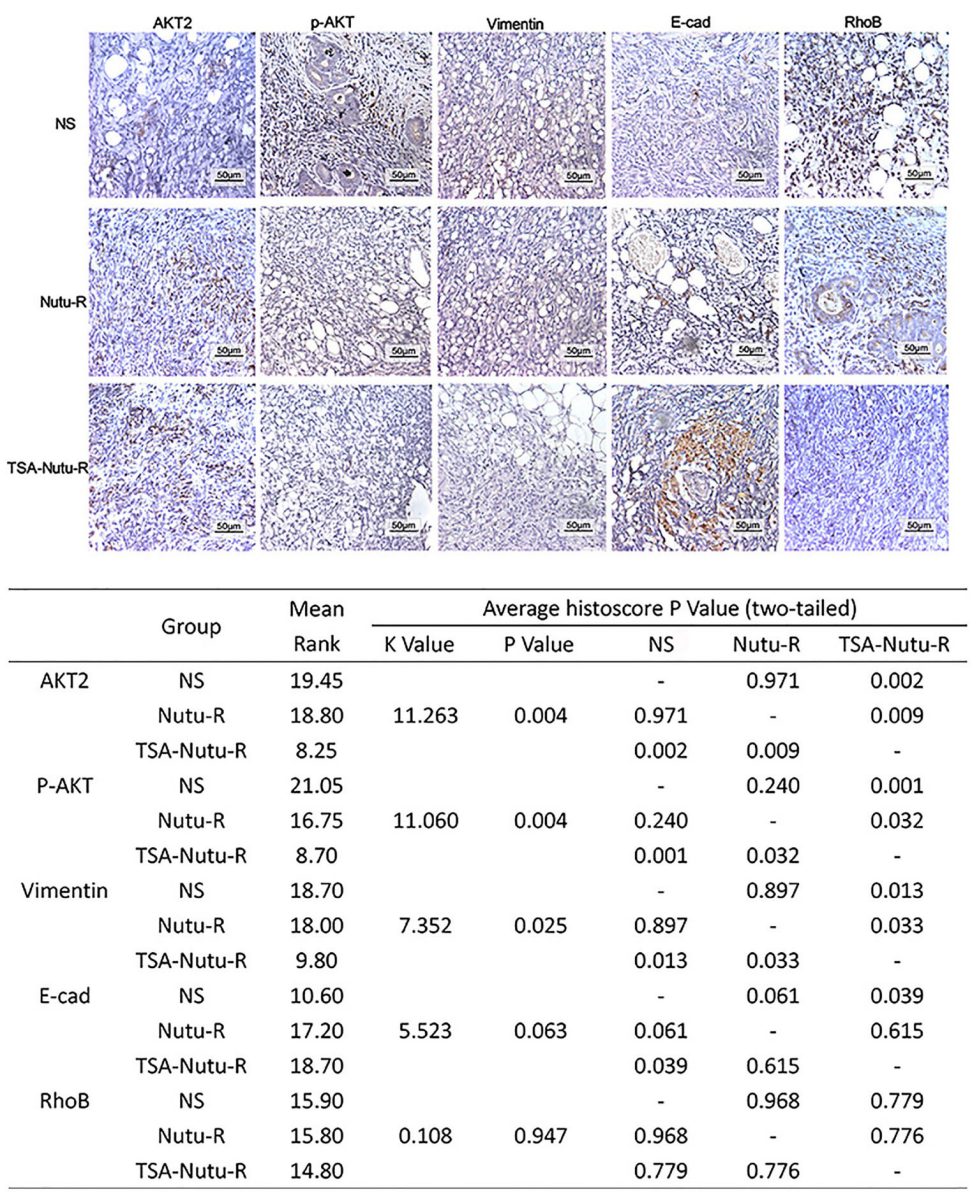
Micrographs taken at 400× magnification, scale bar 50 μm. Expression quantity of RhoB, AKT2/p-AKT, and other signal molecules related to survival in TSA-Nutu-R vaccine group, Nutu-R vaccine group, and control group (NS) (scale bar 50 μm). The table shows the histological score of each group and multiple comparisons by ANOVA.

To explore the reasons for the antitumor effects and prolonged survival, the corresponding signal molecules were detected through immunohistochemistry analysis (Figure 6). The experimental findings indicated that AKT2/p-AKT/RhoB proteins played a crucial role in prolonging the survival cycle of tumor-bearing hosts. Immunohistochemistry analysis demonstrated that the expression of RhoB and AKT2/p-AKT in the TSA-NuTu-R vaccine group was altered compared to the NuTu-R vaccine and control groups. The activation of AKT2/p-AKT/RhoB signaling pathway in OC treated by TSA-NuTu-R vaccine was also evaluated. RhoB gene self-silencing in OC cells was markedly reversed by TSA-NuTu-R vaccine, but p-AKT was remarkably downregulated. Thus, it is speculated that RhoB/AKT2/p-AKT signaling pathway may be involved in this regulatory mechanism.

### Toxicity was not observed in TSA-NuTu-R vaccine-treated rats

No adverse events were observed in gross measures, including behavior, ruffling of fur, lifespan, feeding, or weight loss. No pathological changes were observed in the lung, heart, liver, and kidney, as revealed by microscopic examination. Moreover, the immunized rats had no alveolar enlargement compared to the non-immunized rats (Supplementary Figure S1).

## Discussion

Over the past decades, antitumor vaccines have been extensively studied (19,20). However, the clinical application of tumor vaccines is still uncertain. Besides a few exceptions of melanoma antigen, there is still lack of data about the identity of CTL epitopes and antigenic peptides presented by solid tumors (21). The paucity of vaccines that elicit antitumor immune responses to eliminate tumors may hamper the development of tumor cell-mediated vaccines (22,23). Currently, cancer is also considered as a various gene-driven disease, instead of single gene-driven disease. The genetic profile of a tumor can be revealed through the application of new technologies such as chromosome dissection and microarray chips (24). The tumor first evades the host defenses, and then escapes from the immune system to evolve into a full-blown cancer. The mutations of genes related to immune and apoptotic pathways have been recognized as one of the tumor escape mechanisms. A previous study has shown that epigenetic silencing is a common cause of gene inactivation in tumors similar to gene mutations (25,26).

Infiltration of T cells and other immune effectors in OC have been reported by Haskill et al. (27), and their roles in OC prognosis have become more apparent in recent years. Among 74 patients with a complete clinical response after surgery and platinum-based therapy, the five-year survival rate was more than 70% in patients with infiltrating CD3+ T cells compared to 11.9% in patients without infiltrating T cells (25). Immunotherapeutic approaches, such as adoptive T-cell therapy, antibody treatment (i.e. catumaxomab, pertuzumab, and farletuzumab), and vaccines, have been proposed for the treatment of OC (28). Kryczek et al. (29) reported that an elevated IL-17 level was associated with better prognosis, implying that IL-17-producing Th17 cells have the potential of directly killing tumor cells. In this study, the expression levels of multiple key factors of the immunoregulatory network including cytokines and chemokines induced by various doses of TSA-NuTu-R vaccine were investigated by magnetic bead microarray. Our results showed that IL-17 was also associated with the antitumor effects of TSA-NuTu-R vaccine. This vaccine could induce both therapeutic and protective antitumor immune responses. It has been reported that HDACi can regulate the expression levels of numerous genes (26,30), which is considered as an additional mechanism rather than direct presentation.

The present study demonstrated that HDACi-modified vaccine is effective in both therapeutic and preventative models, and it can trigger innate immunity and induce adaptive immune responses, thereby suppressing the growth and tumorigenesis of OC. Efforts are ongoing to develop novel strategies for tumor cell-mediated vaccines. *In vivo* studies indicated that TSA-NuTu-R vaccine could trigger an effective immune defense that protects the host from tumorigenesis and tumor progression. *In vitro* ELISpot assay verified that TSA-NuTu-R vaccine could trigger immune defense against OC, and further confirmed that the immune activity against this tumor was mainly via IFN-γ and IL-4 pathways. Additionally, NK/T cell-mediated cytotoxicity responses by TSA-NuTu-R vaccine play critical roles in host defenses against tumorigenesis and disease progression.

Our data demonstrated that the TSA-modified OC vaccines could effectively induce NK cell activity to eliminate an MHC class I-deficient tumor. The antibody-dependent cell-mediated cytotoxicity was obviously induced after TSA-NuTu-R vaccination. Notably, the anti-serum obtained from the TSA-NuTu-R vaccine group regulated tumor suppression in the OC rat model via adoptive cell transfer therapy. Moreover, antitumor CD8+ CTLs were effectively reactivated through CD4+ T cells after TSA-NuTu-R vaccination. The antitumor activity could lead to integrated immunoregulation, such as accentuation of CTL activity, secretion of Th1-type cytokine (IFN-γ), induction of antibody-dependent cellular cytotoxicity, enhancement of adaptive T-cell responses, and activation of NK cells. Hence, the TSA-NuTu-R vaccine may serve as a promising antitumor vaccination strategy, which triggers innate immunity and induces adaptive immune responses, thereby suppressing the growth and tumorigenesis of OC.

One of the methods used to produce whole tumor vaccines is using a lethal dose of ultraviolet (UV) irradiation to generate whole apoptotic tumor cells. The asymmetry of the cell plasma membrane phospholipids is lost, exposing phosphatidylserine (31,32). X-ray irradiation could lead to phosphorylation and membrane translocation of calreticulin and further enhance immunogenicity (33). In our previous research, we have explored various levels of irradiation to determine the appropriate irradiation dose for tumor vaccine (34). The tumor cells only irradiated by X-ray as the control group in the study still showed a slight immune effect, indicating that the irradiated cells may also cause a certain level of immune antigen exposure, but the efficacy is not as significant as that of the TSA-modified tumor vaccine.

In our previous study (14), epigenetic modulation of the tumor suppressor gene RhoB was evaluated in OC cells treated with TSA. Some other studies have demonstrated that RhoB promotes the nuclear entry of phosphorylated Akt, or impedes the nuclear export of phosphorylated Akt after its delivery (35). Jiang et al. (36) demonstrated that Ras could downregulate the expression of RhoB through a PI3K/Akt-dependent mechanism but not a Mek-dependent mechanism (37). In this study, we also found that the expression of RhoB and AKT2 was upregulated, while p-AKT was downregulated in the TSA-NuTu-R vaccine group compared to the control and other experimental groups. The above data suggested that the epigenetic-modified tumor vaccine may activate the AKT signaling pathway to suppress immune responses in OC tissues.

### Conclusions

In summary, this study showed that altering the acetylation status of chromatin may enhance the effectiveness of a tumor vaccine. It is worth noting that the rats had no significant adverse events after immunization with the proposed vaccine. However, there may be a huge challenge in applying this vaccine in the treatment of OC. Therefore, future studies are warranted to assess the synergistic effects of this vaccine and other therapeutic agents, in terms of immune responses and specific immunological targets.

## Supplementary Material

Click here to view [pdf].

## References

[1] Cobec IM, Sas I, Moatar AE, Moleriu L, Rempen A (2021). Ovarian cancer health politics in Romania and Germany: a
comparative study. Exp Ther Med.

[2] Morand S, Devanaboyina M, Staats H, Stanbery L, Nemunaitis J (2021). Ovarian cancer immunotherapy and personalized
medicine. Int J Mol Sci.

[3] Kandalaft LE, Motz GT, Duraiswamy J, Coukos G (2011). Tumor immune surveillance and ovarian cancer: lessons on immune
mediated tumor rejection or tolerance. Cancer Metastasis Rev.

[4] Shen S, Wang G, Zhang R, Zhao Y, Yu H, Wei Y (2019). Development and validation of an immune gene-set based prognostic
signature in ovarian cancer. EBiomedicine.

[5] Bogani G, Lopez S, Mantiero M, Ducceschi M, Bosio S, Ruisi S (2020). Immunotherapy for platinum-resistant ovarian
cancer. Gynecol Oncol.

[6] Grunewald T, Ledermann JA (2017). Targeted therapies for ovarian cancer. Best Pract Res Clin Obstet Gynaecol.

[7] Yang C, Xia BR, Zhang ZC, Zhang YJ, Lou G, Jin WL (2020). Immunotherapy for ovarian cancer: adjuvant, combination, and
neoadjuvant. Front Immunol.

[8] Hinchcliff E, Jazaeri AA (2019). Sunset, or dawn of a new age for ovarian cancer vaccine
therapy?. Gynecol Oncol.

[9] Mazumder S, Swank V, Dvorina N, Johnson JM, Tuohy VK (2022). Formulation of an ovarian cancer vaccine with the squalene-based
AddaVax adjuvant inhibits the growth of murine epithelial ovarian
carcinomas. Clin Exp Vaccine Res.

[10] Voelker R (2018). Pursuing an effective ovarian cancer vaccine. Jama.

[11] Xiang SD, Gao Q, Wilson KL, Heyerick A, Plebanski M (2015). Mapping T and B cell epitopes in sperm protein 17 to support the
development of an ovarian cancer vaccine. Vaccine.

[12] Guo J, De May H, Franco S, Noureddine A, Tang L, Brinker CJ (2022). Cancer vaccines from cryogenically silicified tumour cells
functionalized with pathogen-associated molecular patterns. Nat Biomed Eng.

[13] Srivatsan S, Patel JM, Bozeman EN, Imasuen IE, He S, Daniels D (2014). Allogeneic tumor cell vaccines: the promise and limitations in
clinical trials. Hum Vacc Immunother.

[14] Liu Y, Song N, Ren K, Meng S, Xie Y, Long Q (2013). Expression loss and revivification of RhoB gene in ovary
carcinoma carcinogenesis and development. Plos One.

[15] Mariadason JM, Corner GA, Augenlicht LH (2000). Genetic reprogramming in pathways of colonic cell maturation
induced by short chain fatty acids: comparison with trichostatin A,
sulindac, and curcumin and implications for chemoprevention of colon
cancer. Cancer Res.

[16] Cacan E, Greer SF, Garnett-Benson C (2015). Radiation-induced modulation of immunogenic genes in tumor cells
is regulated by both histone deacetylases and DNA
methyltransferases. Int J Oncol.

[17] Khan AN, Tomasi TB (2008). Histone deacetylase regulation of immune gene expression in tumor
cells. Immunol Res.

[18] Khan AN, Magner WJ, Tomasi TB (2007). An epigenetic vaccine model active in the prevention and
treatment of melanoma. J Transl Med.

[19] Liu Y, Yan B, Wang Z, Zhu H, Yin X, Wang K (2020). Design, synthesis, and preliminary immunological studies of
MUC1-based antitumor vaccines adjuvanted with R- and S-FSL-1. ACS Med Chem Lett.

[20] Vasquez M, Tenesaca S, Berraondo P (2017). New trends in antitumor vaccines in melanoma. Ann Transl Med.

[21] Du JJ, Zou SY, Chen XZ, Xu WB, Wang CW, Zhang L (2019). Liposomal antitumor vaccines targeting mucin 1 elicit a
lipid-dependent immunodominant response. Chem Asian J.

[22] Accolla RS, Buonaguro L, Melief C, Rammensee HG, Bassani-Sternberg M (2019). Editorial: novel strategies for anti-tumor
vaccines. Front Immunol.

[23] Rammensee HG, Loffler MW, Walz JS, Bokemeyer C, Haen SP, Gouttefangeas C (2020). Tumor vaccines-therapeutic vaccination against cancer [in
German]. Internist (Berl).

[24] Chen J, Zhang H, Zhou L, Hu Y, Li M, He Y (2021). Enhancing the efficacy of tumor vaccines based on immune evasion
mechanisms. Front Oncol.

[25] Lettini AA, Guidoboni M, Fonsatti E, Anzalone L, Cortini E, Maio M (2007). Epigenetic remodelling of DNA in cancer. Histol Histopathol.

[26] Tomasi TB, Magner WJ, Khan AN (2006). Epigenetic regulation of immune escape genes in
cancer. Cancer Immunol Immun.

[27] Haskill S, Becker S, Fowler W, Walton L (1982). Mononuclear-cell infiltration in ovarian cancer. I.
Inflammatory-cell infiltrates from tumour and ascites
material. Br J Cancer.

[28] Leon E, Ranganathan R, Savoldo B (2020). Adoptive T cell therapy: boosting the immune system to fight
cancer. Semin Immunol.

[29] Kryczek I, Banerjee M, Cheng P, Vatan L, Szeliga W, Wei S (2009). Phenotype, distribution, generation, and functional and clinical
relevance of Th17 cells in the human tumor environments. Blood.

[30] Sheyhidin I, Hasim A, Zheng F, Ma H (2014). Epigenetic changes within the promoter regions of antigen
processing machinery family genes in Kazakh primary esophageal squamous cell
carcinoma. Asian Pac J Cancer Prev.

[31] Chiang CL, Benencia F, Coukos G (2010). Whole tumor antigen vaccines. Semin Immunol.

[32] Chiang CL, Coukos G, Kandalaft LE (2015). Whole tumor antigen vaccines: where are we?. Vaccines (Basel).

[33] Obeid M, Panaretakis T, Joza N, Tufi R, Tesniere A, van Endert P (2007). Calreticulin exposure is required for the immunogenicity of
gamma-irradiation and UVC light-induced apoptosis. Cell Death Differ.

[34] Zhang YN, Duan XG, Zhang WH, Wu AL, Yang HH, Wu DM (2016). Antitumor activity of pluripotent cell-engineered vaccines and
their potential to treat lung cancer in relation to different levels of
irradiation. Onco Targets Ther.

[35] Adini I, Rabinovitz I, Sun JF, Prendergast GC, Benjamin LE (2003). RhoB controls Akt trafficking and stage-specific survival of
endothelial cells during vascular development. Gene Dev.

[36] Jiang K, Sun J, Cheng J, Djeu JY, Wei S, Sebti S (2004). Akt mediates Ras downregulation of RhoB, a suppressor of
transformation, invasion, and metastasis. Mol Cell Biol.

[37] Bousquet E, Maziàres J, Privat M, Rizzati V, Casanova A, Ledoux A (2009). Loss of RhoB expression promotes migration and invasion of human
bronchial cells via activation of AKT1. Cancer Res.

