# Supplementary material for: Trichostatin A-modified vaccine provides superior protection against ovarian cancer formation and development
Source: Braz J Med Biol Res. 2024 May 17;57:e12874. doi: 10.1590/1414-431X2024e12874 (PMC11101164; doi:10.1590/1414-431X2024e12874)

Figure S1. Microphotographs of organ tissues (HE) of TSA-Nutu-R vaccine group, Nutu-R vaccine group, and control group (scale bar 200 μm). No significant changes were observed. TSA: trichostatin A.

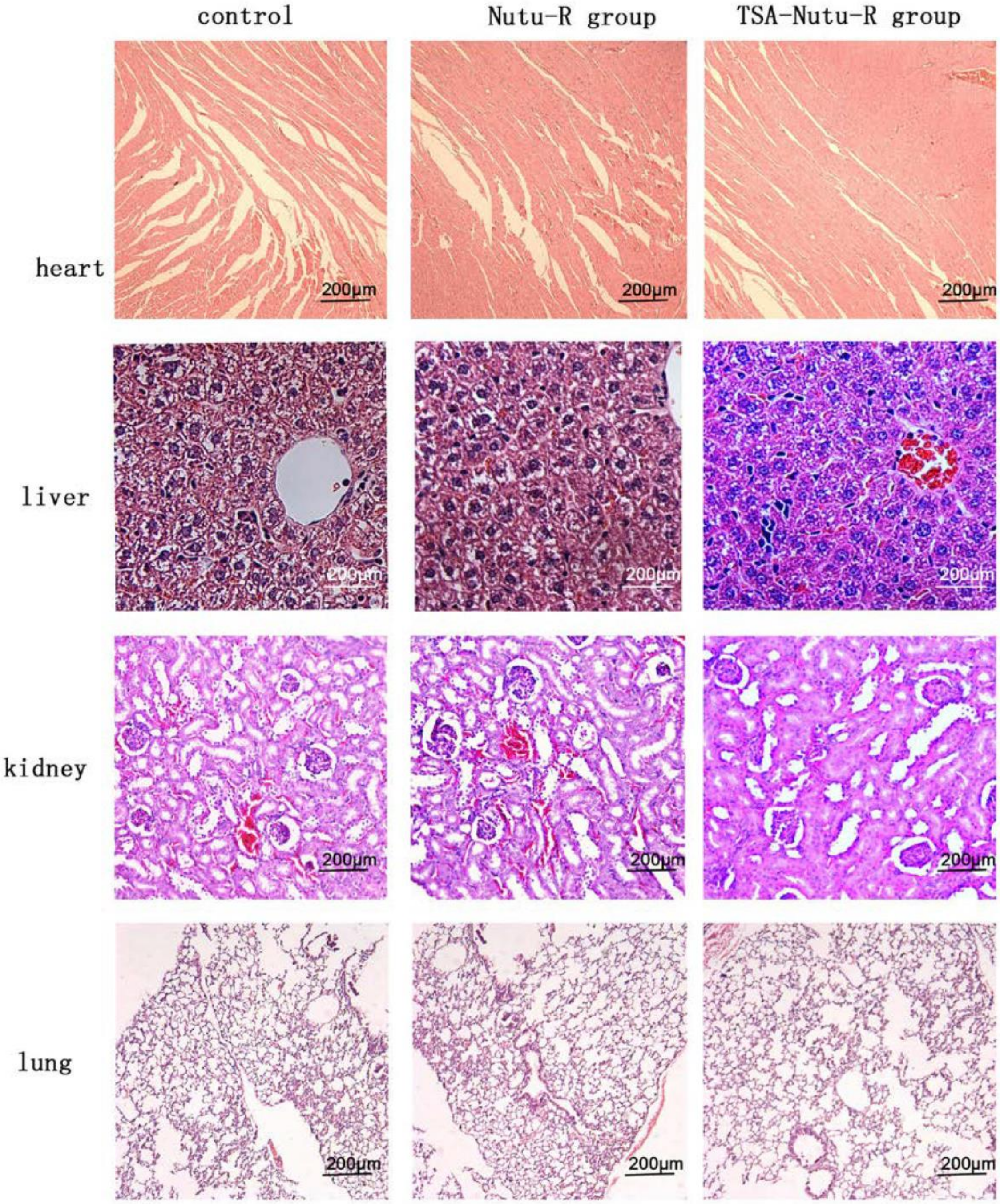

Supplement: Supplementary file 1 [file 1414-431X-bjmbr-57-e12874-suppl.pdf]
